# Association of Severe Malaria Outcomes with Platelet-Mediated Clumping and Adhesion to a Novel Host Receptor

**DOI:** 10.1371/journal.pone.0019422

**Published:** 2011-04-29

**Authors:** Alfredo Mayor, Abdul Hafiz, Quique Bassat, Eduard Rovira-Vallbona, Sergi Sanz, Sónia Machevo, Ruth Aguilar, Pau Cisteró, Betuel Sigaúque, Clara Menéndez, Pedro L. Alonso, Chetan E. Chitnis

**Affiliations:** 1 Barcelona Centre for International Health Research (CRESIB), Hospital Clínic/Institut d'Investigacions Biomèdiques August Pi i Sunyer (IDIBAPS), Universitat de Barcelona, Barcelona, Spain; 2 Centro de Investigação em Saúde de Manhiça (CISM), Maputo, Mozambique; 3 Consorcio de Investigación Biomédica de Epidemiología y Salud Pública (CIBERESP), Madrid, Spain; 4 International Centre for Genetic Engineering and Biotechnology (ICGEB), New Delhi, Índia; Agency for Science, Technology and Research (A*STAR), Singapore

## Abstract

**Introduction:**

Severe malaria has been attributed partly to the sequestration of *Plasmodium falciparum*-infected erythrocytes (IEs) in the microvasculature of vital host organs. Identification of *P. falciparum* cytoadherence phenotypes that are associated with severe malaria may lead to the development of novel strategies against life-threatening malaria.

**Methods and Findings:**

Forty-six *P. falciparum* isolates from Mozambican children under 5 years of age with severe malaria (cases) were examined and compared to 46 isolates from sex and age matched Mozambican children with uncomplicated malaria (controls). Cytoadherence properties such as platelet-mediated clumping, rosetting and adhesion to purified receptors (CD36, ICAM1 and gC1qR), were compared between these matched pairs by non-parametric tests. The most common clinical presentation associated with severe malaria was prostration. Compared to matched controls, prevalence of platelet-mediated clumping was higher in cases (P = .019), in children presenting with prostration (P = .049) and in children with severe anaemia (P = .025). Prevalence of rosetting and gC1qR adhesion were also higher in isolates from cases with severe anemia and multiple seizures, respectively (P = .045 in both cases), than in controls.

**Conclusions:**

These data indicate a role for platelet-mediated clumping, rosetting and adhesion to gC1qR in the pathogenesis of severe malaria. Inhibition of these cytoadherence phenotypes may reduce the occurrence or improve the prognosis of severe malaria outcomes.

## Introduction

*Plasmodium falciparum* causes approximately 500 million clinical episodes of malaria per year [1]. A small fraction (1–2%) of these malaria attacks develop into severe malaria, specially in children less than 5 years of age [2]. Case fatality rates for severe malaria remain unacceptably high even after effective anti-malarial drugs are administered [3]. Several ancillary treatments have been tested in combination with anti-malarial drugs [4]–[6] but, to date, there is little evidence to recommend their use. A greater understanding of the pathogenesis of severe malaria is essential for the rational development of novel interventions.

The following two theories are proposed to explain the patho-physiological mechanisms leading to severe malaria. The cytokine-based hypothesis suggests that inflammatory mediators such as cytokines and nitric oxide released by host cells following exposure to malaria ‘toxins’ have a major role in the onset of the pathology of severe malaria [7]. In contrast, the mechanical theory suggests that severe malaria is attributable to excessive sequestration of *P. falciparum*-infected erythrocytes (IEs) in the microvasculature of vital host organs leading to mechanical obstruction of blood flow, hypoxia, tissue damage and, ultimately, organ failure [8]. Sequestration of parasites is suggested to be mediated through the adherence of mature forms of *P. falciparum* IEs to host receptors expressed on the endothelium lining host capillaries, on uninfected erythrocytes to form ‘rosettes’ [9] and on platelets to form platelet-mediated clumps [10]. The following observations support cytoadherence as the main process leading to severe malaria. First, among all human malaria parasites, only *P. falciparum*, which is responsible for most of the malaria related deaths, is known to sequester [8]. Second, autopsy studies have suggested that parasite sequestration plays an important role in cerebral malaria (CM) [11], [12]. Third, estimation of the sequestered parasite biomass based on plasma histidine-rich protein-2 has shown that disease severity increases with increasing parasite sequestration [13]. Finally, organ-specific accumulation of distinct *P. falciparum* variant types suggests that IEs can exhibit preferential adhesion in the body [14].

*In vitro* analysis of the cytoadherence phenotypes of *P. falciparum* isolates derived from infected individuals has shown a wide range of binding affinities to numerous host receptors such as intercellular adhesion molecule ICAM-1 [15], CD36 [16], chondroitin sulphate A (CSA) [17], gC1qR [18] and CR1 [19]. However, the patho-physiological significance of these cytoadherence phenotypes remains unclear as associations with severe malaria or specific signs of severity have been found in some studies [10], [20]–[26] but not in others [24], [27]–[29]. Moreover, some cytoadherence phenotypes, such as adhesion to CD36, have been associated with uncomplicated malaria [22], [24], [29], suggesting a protective role in the context of severe disease [30], [31].

Here we have examined the relationship between specific adhesion phenotypes of *P. falciparum* isolates, namely, adhesion to ICAM1, CD36 and gC1qR, rosetting and platelet-mediated clumping, and disease severity in children less than 5 years of age residing in a malaria-endemic area of Mozambique.

## Results

Among the 142 parasite isolates included in the study, 111 (78%) matured to trophozoite stages. The proportion of isolates that did not recover from cryopreservation was similar for parasite isolates collected from children with severe (n = 19, 27%) or uncomplicated malaria (n = 12, 17%, p = 0.223). Parasitemia at thawing was higher for isolates from children with severe malaria (7.3%, interquartile range [IQR] [2.2–12.4]) than in children with uncomplicated malaria (3.5%, IQR [1.7–5.9], p = 0.011). Of the 111 isolates that matured to trophozoite stage, 46 matched case-control pairs of parasites could be used for analysis. The characteristics of the 92 patients that provided the parasite isolates used are summarized in Table 1. Paired analysis showed that children with severe and uncomplicated malaria were not significantly different in the prevalence of previous malaria treatment, days of fever, history of cough or vomiting, platelet count and multiplicity of infection (MOI) at recruitment, although parasitemia was higher in severe malaria cases than controls. Among the 46 cases of severe malaria, 19 (41%) had a single criteria of malaria severity, 15 (33%) had two, and 12 (26%) three or more. Of the 19 cases with a single criterion of malaria severity, 13 had prostration, 5 had multiple seizures and 1 had severe anemia. Prostration was the most prevalent of the severe malaria signs (Table 1), followed by acute respiratory distress (ARD), severe anemia and multiple seizures. Only 3 (6%) patients had CM and only 2 (4%) had hypoglycaemia. Two severe malaria patients died, yielding a case fatality rate of 2.2%. Final outcome could not be ascertained for two severe patients, as one absconded in a very serious condition and the other was transferred to Maputo's Central Hospital.

**Table 1:** Clinical and biological characteristics of the study participants at the time of admission.

	Severe malaria	Non-severe malaria	
Patient's characteristics	n = 46	n = 46	*P* a
**Demographic data**			
Age (months)	32.5 (17–43)	32 (17–42)	.871
Males, n (%)	31 (67.4)	31 (67.4)	1.000
**Pre-treatment**			
Anti-pyretic, n (%)	3 (6)	0 (0)	.083
Antibiotic, n (%)	2 (4%)	1 (2)	.564
Antimalarial, n (%)	1 (2)	0 (0)	.317
**Physical findings**			
Temperature (°C)	38.6 (37.9–39.2)	38.2 (36.8–39.6)	.880
Weight (Kg)	11.4 (9.4–13.2)	11.9 (10.0–12.9)	1.000
Splenomegaly, n (%)	23 (50.0)	10 (21.7)	.067
Hepatomegaly, n (%)	8 (17.4)	2 (43.5)	.033
Previous days of fever	1 (1–2)	1 (1–2)	.860
Previous days of cough	1 (0.5–1)	1 (0–1)	.250
Previous days of vomits	0 (0–0)	1 (1–1)	.250
**Laboratory parameters**			
Microscopic parasitaemia (10^3^/µL)	53.5 (28.1–129.4)	40.5 (18.2–73.7)	.007
Multiplicity of infection	3 (3–4)	3 (2–4)	.405
Packed cell volume (%)	28 (16–32)	32 (28–34)	.019
Platelets (10^9^/L)	116 (76–184)	143 (92–181)	.164
Glucose (mM)	6.0 (5.2–7.0)	5.9 (5.4–6.8)	.532
White blood cells (10^9^/L)	10.1 (7.4–12.7)	5.9 (5.4–6.8)	.059
Lymphocytes (%)	31.4 (21.3–43.1)	37.4 (23.6–50.6)	.014
Neutrophiles (%)	63.9 (46.2–71.1)	56.5 (38.3–65.9)	.065
Creatinin (µ/L)	35 (30–38)	34 (30–38)	.542
Bilirubin (µM)	24 (17–38)	15 (10–24)	.009
ALT (µ/L)	33 (21–39)	25 (18–35)	.371
Lactate (mM)	3.7 (2.3–5.0)	2.4 (1.9–3.7)	.009
**Severe malaria syndromes**			
Prostration, n (%)	37 (80)	-	
ARD, n (%)	19 (41)	-	
Severe anemia, n (%)	14 (30)	-	
Multiple seizures, n (%)	13 (28)	-	
Cerebral malaria, n (%)	3 (6)	-	
Hypoglycemia, n (%)	2 (4)	-	

All continuous data is presented as the Median (Interquartile range).

ALT: Alanine aminotransferase; ARD: Acidosis or respiratory distress; IQR: Interquartile Range.

a , McNemar's chi-squared (categorical) and Singtest (continuous).

Isolates were considered positive for rosetting if frequency of rosettes was higher than 2% [32] and for platelet-mediated clumping if frequency of clumps was higher in presence of platelets than in buffer-control. Adhesion to host receptors was determined by measuring binding to purified receptors coated onto Petri dishes. Non-specific background adhesion was determined by measuring binding to Duffy antigen coated on Petri dishes. Based on the mean number of IEs bound to Duffy antigen (1.91per mm^2^; standard deviation 8.78), we established the cut-off for positive binding to endothelial host receptors as 19.5 per mm^2^ (mean+ 2 standard deviations).

I was doing it and I will do my best to finish Sixty out of 92 *P. falciparum* isolates tested (65%) formed clumps in presence of platelets. Among those isolates showing platelet-mediated clumping, patient's platelet count decreased with increasing frequency of clumps (rho = −0.258, p = 0.051). No correlation was found between frequency of clumps and parasitemia at thawing (rho = 0.09, P = 0.457). Results from platelet-mediated clumping assays performed without adjusting parasitemia correlated well with results from the same assay performed with parasitemias adjusted to 1% (n = 72, rho = 0.754, p<0.001). Both assays gave similar results in terms of prevalence of clumping (unadjusted: 44/72 [61%]; adjusted: 47/72 [65%]; p = 0.405), although medians for frequency of clumping were slightly higher in assays with unadjusted parasitemias (1.9%, IQR [0.0–6.4]) compared to assays performed at 1% parasitemia (1.6%, IQR [0.0–13.7]; p = 0.006).

Adhesion to CD36 was the most frequent cytoadherence phenotype among *P. falciparum* isolates (80/92, 87%), followed by platelet-mediated clumping (60/92, 65%), adhesion to ICAM1 (41/92, 45%), to gC1qR (39/92, 42%) and rosetting (31/86, 36%). Among the isolates showing cytoadherence, median adhesion (parasites per mm^2^) was highest for CD36 (179, IQR [95–332]), followed by gC1qR (60, IQR [45–155]) and ICAM1 (55, IQR [35–105]). Median frequency of rosetting and platelet-mediated clumping was 3.0% (IQR [1.9–5.3]) and 7.2% (IQR [1.9–23.3]), respectively.

Cytoadherence phenotypes were compared between isolates from children presenting with severe malaria or specific severe malaria syndromes and their matched controls. Tables 2 and 3 show the prevalence and median levels of cytoadherence phenotypes respectively. Although not statistically significant, intensities of adhesion to CD36 were always lower in isolates from children with severe malaria than in their matched controls (Table 3). Prevalence and level of platelet-mediated clumping was significantly higher in isolates from severe cases compared to matched controls, both when the assays were conducted with unadjusted parasitemias (Table 2 and 3) and when parasitemias were adjusted to 1% (Table S1).

**Table 2:** Number (%) of isolates showing cytoadherence in children with severe and non-severe malaria.

Clinical malaria	Rosetting	PM-Clumping	CD36	ICAM1	gC1qR	Duffy
**Uncomplicated (n = 46)**	15^a^ (34.9)	24 (52.2)	39 (84.8)	18 (39.1)	17 (36.9)	2 (4.3)
**Severe (n = 46)**	16^a^ (37.2)	36 (78.3)	41 (89.1)	23 (50.0)	22 (47.8)	1 (2.2)
***P***	.808	**.019**	.527	.297	.297	.564
**Uncomplicated (n = 37)**	13^a^(38.2)	20 (54.0)	31 (83.8)	14 (37.8)	13 (35.1)	1 (2.7)
**Prostration (n = 37)**	13^a^ (38.2)	29 (78.5)	33 (89.2)	18 (48.7)	17 (45.9)	1 (2.7)
***P***	1.000	**.049**	.479	.355	.355	1.000
**Uncomplicated (n = 19)**	5^b^ (27.8)	11 (57.9)	17 (89.5)	9 (47.4)	9 (47.4)	1 (5.3)
**ARD (n = 19)**	7^b^ (38.9)	15 (78.9)	17 (89.5)	8 (42.1)	5 (26.3)	0 (0.0)
***P***	.317	.157	1.000	.781	.205	.317
**Uncomplicated (n = 14)**	2^b^ (15.4)	8 (57.1)	13 (92.8)	6 (42.9)	5 (35.7)	2 (14.3)
**Severe anaemia (n = 14)**	6^b^ (46.2)	13 (92.8)	12 (85.7)	6 (42.8)	6 (42.9)	0 (0.0)
***P***	**.045**	**.025**	.564	1.000	.739	.157
**Uncomplicated (n = 13)**	4 (30.8)	5 (38.5)	8 (61.5)	5 (38.5)	2 (15.4)	0 (0.0)
**Multiple seizures (n = 13)**	4 (30.8)	9 (84.6)	11 (84.6)	9 (69.2)	6 (46.2)	1 (7.7)
***P***	1.000	.206	.179	.157	**.045**	0.317

a : 3 missing;

b : 1 missing.

PM-Clumping: Platelet-mediated clumping (unadjusted); ARD: Acidosis and/or respiratory distress.

**Table 3:** Median (interquartile range) intensities of cytoadherence phenotypes by clinical malaria manifestation.

Clinical malaria	Rosetting	PM-Clumping	CD36	ICAM1	gC1qR
**UM (n = 46)**	0.6^a^ (0.0; 2.6)	0.2 (0.0; 8.7)	178.1 (50.0; 405.0)	2.5 (0.0; 40.0)	0.0 (0.0; 45.0)
**SM (n = 46)**	1.0^a^ (0.2; 4.6)	5.1 (0.4; 20.2)	123.4 (50.0; 245.0)	15 (0.0; 55.0)	8.5 (0.0; 70.0)
***P***	.627	**.032**	1.000	.499	.229
**UM (n = 37)**	0.6^a^ (0.0; 3.0)	0.2 (0.0; 8.7)	171.6 (50.0; 405.0)	0.0 (0.0; 45.0)	0.0 (0.0; 48.6)
**P (n = 37)**	1.0^a^ (0.2; 4.0)	4.7 (0.4; 20.4)	127.1 (50.0; 245.0)	10.0 (0.0; 55.0)	0.0 (0.0; 75.0)
***P***	.855	**.041**	1.000	.701	.327
**UM (n = 19)**	0.2^b^ (0.0; 2.2)	2.6 (0.0; 16.9)	275.0 (125.0; 533.4)	15.0 (0.0; 54.2)	0.0 (0.0; 55.0)
**ARD (n = 19)**	1.1^b^ (0.0; 5.3)	6.3 (0.4; 20.2)	110.0 (44.9; 175.0)	0.0 (0.0; 35.0)	0.0 (0.0; 75.0)
***P***	.791	.323	.167	.803	.267
**UM (n = 14)**	0.9^b^ (0.0; 2.7)	0.3 (0.0; 11.6)	178.1 (52.5; 423.5)	0.0 (0.0; 37.9)	0.0 (0.0; 45.0)
**SM (n = 14)**	2.0^b^ (0.2; 7.7)	6.6 (2.0; 15.5)	75.0 (45.0; 175.0)	0.0 (0.0; 47.7)	2.5 (0.0; 55.0)
***P***	.065	.092	.424	.549	1.000
**UM (n = 13)**	0.2 (0.0; 2.2)	2.6 (0.0; 16.9)	275.0 (125.0; 533.4)	15.0 (0.0; 54.2)	0.0 (0.0; 55.0)
**MS (n = 13)**	1.0 (0.2; 2.2)	2.5 (0.0; 7.0)	120.3 (45.0; 255.0)	22.4 (0.0; 51.6)	12.0 (0.0; 50.0)
***P***	1.000	.774	.774	.387	.125

a : 3 missing;

b : 1 missing.

PM-Clumping: Platelet-mediated clumping; UM: Uncomplicated malaria; SM: severe malaria; P: Prostration; ARD: Acidosis and/or respiratory distress; SA: Severe anemia; MS: Multiple seizures.

P-values for cytoadherence to Duffy receptors are as follows: 0.453 for SM; 1.000 for P, D and MS; and 0.625 for SA.

For the rest of the cytoadhesion phenotypes, a uniform trend towards higher levels of cytoadherence among parasites isolated from children with severe malaria was observed, although these differences were not statistically significant. Platelet-mediated clumping assessed at unadjusted parasitemias was also higher for those parasites isolated from children with prostration and severe anemia, as compared to their matched controls (Table 2 and 3). Similar results were found when the assay was conducted at 1% of parasitemia, although differences reached statistical significance for ARD but not for prostration (Table S1). These differences probably reflect different sample sizes (46 pairs for unadjusted and 36 for adjusted parasitemias). Adhesion to gC1qR was found to be significantly higher in isolates from children with multiple seizures compared to matched controls (Table 2). Rosetting was also higher among isolates from children with severe anemia (Table 2 and 3). Adhesion phenotypes of isolates from CM and hypoglycemia cases could not be statistically compared to matched controls due to low numbers (n = 3 and 2, respectively).

## Discussion

The results of this case-control study suggest that platelet-mediated clumping is associated with severe malaria. Moreover, to our knowledge, this study reports for the first time that platelet-mediated clumping is associated with prostration, and that adhesion to the recently identified cytoadherence receptor gC1qR [18] is associated with multiple seizures, one of the criteria that defines severe malaria.

The 46 patients with severe falciparum malaria participating in this study are representative of this condition in Manhiça (Mozambique) [33], which is characterized by a high prevalence of prostration, severe anemia and ARD, and a lower prevalence of CM. Malaria-associated case fatality rates were slightly lower than previously reported [33], possibly due to the small number of patients and intensive follow-up. The adequate likelihood of malaria being the principal cause of disease was guaranteed by using a minimum parasitaemia threshold (500 parasites/µl) with a proven high sensitivity/specificity balance [34] to enroll the children in the study, as well as the exclusion from the study of those cases with concomitant bacterial infections. Furthermore, no difference between severe malaria cases and non-severe controls was found in the prevalence of previous malaria treatment or the number of days with symptoms of malaria before arrival to hospital, suggesting that severity was not due to a longer progression of the disease among cases.

In accordance with previous studies from other sites [10], [18], [21]–[23], [27]–[29], *P. falciparum* isolates infecting Mozambican children were characterized by their predominant adhesion to CD36 (87%), followed by platelet-mediated clumping (65%), adhesion to ICAM1 (45%), to gC1qR (42%) and rosetting (36%). It could be argued that cryopreservation of parasites may reduce the adhesive ability of IEs, although it has been reported that it does not alter the amount of parasite proteins on the surface of IEs as assessed by flow cytometry [35]. Platelet-mediated clumping was the most prominent cytoadherence phenotype associated with severe malaria in this study. This finding was observed when the clumping data was analyzed in terms of prevalence and frequency of clumps, and also when the assays were conducted at both adjusted and unadjusted parasitemias. These results show that both assays correlated fairly well (rho = 0.754), probably due to the fact that children with low density parasitemias (<500 parasites/µl of blood) were not recruited into the study. The association between severe malaria and platelet-mediated clumping was similar when the analyses were based on data from assays conducted with or without adjusted parasitemias (1%). This study thus provides clear evidence for association of platelet-mediated clumping with severe malaria.

Since pathogenesis may be quite different among the syndromes used to define severe malaria, we have also analyzed cytoadherence separately for each syndrome. Prostration, the most prevalent severe malaria presentation in this study population, was found to be associated with platelet-mediated clumping. Multiple seizures were observed to be associated with adhesion to gC1qR. Seizures are frequent among severe malaria cases. They may occur as part of CM or independently [36], and have been associated with increased neuro-cognitive deficits and mortality [37], [38], suggesting neurological involvement [39]. It is possible that IEs preferentially sequester in the brain using gC1qR, which is expressed on brain endothelial cells [18]. Local obstruction of blood flow may lead to the manifestation of multiple seizures. However, adhesion to ICAM1, which is also expressed on brain endothelial cells [18], was not associated with seizures in this study. Alternatively, engagement of gC1qR by sequestered parasite ligands may reduce the local availability of gC1qR for normal physiological functions, such as inhibition of the complement cascade [40]. Reduced availability of gC1qR may result in over-activation of complement cascade, which may evoke seizures [41]. The role of adhesion to ICAM1, whichhas been previously implicated in the pathogenesis of CM [24], and of gC1qR on CM could not be evaluated as there were only 3 cases of CM among study patients.

Severe anemia was associated with higher platelet-mediated clumping and rosetting, suggesting a role for these cytoadherence phenotypes in the etiology of malaria-related anemia. Destruction of uninfected erythrocytes attached to IEs in rosettes or down regulation of erythrocyte production by abnormal erythrocyte aggregates might also contribute to severe anemia. The role of platelet-mediated clumping in severe anemia is unclear and requires further investigation. If platelet-mediated clumping is found to have a causative role in severe anemia, novel anti-adhesion interventions that prevent platelet-mediated clumping might have an impact on reducing severe anemia in malaria endemic areas. Such interventions might be of public health relevance, as severe anemia is an important contributor to malaria-related morbidity and mortality in children residing in sub-Saharan Africa [42] and the most important risk factor for death in Mozambican children younger than 8 months with malaria [33].

Role of adhesion to CD36 in the physiopathology of severe malaria is still a subject of debate, as some studies have found higher binding in isolates infecting children with severe malaria [43], [44] and others in parasites infecting children with non-severe malaria [22], [24], [29]. Although not statistically significant, these results show a consistent trend towards higher levels of adhesion to CD36 in parasites isolated from children with uncomplicated malaria than from children with severe malaria, suggesting that adherence to CD36 might be an indicator of a less pathogenic infection. Observations from this study suggest that anti-adhesion therapies should be considered with caution, as blocking adhesion to CD36 may potentially lead to selection of parasites with affinities for other receptors which might be detrimental to the host.

In summary, the results of this case-control study support the hypothesis that cytoadherence properties of *P. falciparum* may be an important virulence factor. The potential for inhibiting or reversing platelet-mediated clumping and adhesion to gC1qR with antibodies or receptor analogs could improve severe malaria outcomes and should be actively explored.

## Materials and Methods

### Ethics statement

The study protocol was approved by the National Ethics Review Committee of Mozambique and the Ethics Review Committee of the Hospital Clinic of Barcelona.

### Study area

The study took place in Manhiça District, southern Mozambique. The study area, which is characterized by perennial malaria transmission with some seasonality, has been extensively described elsewhere [45]. Malaria in Manhiça is primarily caused by *P. falciparum* infections with an average entomological inoculation rate of 38 infective bites per person per year in 2002 [46]. Respiratory distress and anaemia, together with the clinical sign of prostration, are the most prevalent presentations of severe malaria, with the majority of cases occurring in younger children, as opposed to coma, which is infrequent and slightly shifted to older children [33].

### Patients

Between April and November 2006, a sex and age (+/−3 months) matched case-control study was conducted, the cases being children under five years of age with severe malaria (n = 71) and controls being outpatient children with non-severe malaria (n = 71). Children attending the Manhiça District Hospital with a primary clinical diagnosis of *P. falciparum* malaria were recruited into the study after written informed consent was given by their parents or guardians. Blood smears were stained with Giemsa and examined by microscopy according to quality-controlled procedures [47]. Clinical malaria was defined as the presence of fever (axillary temperature≥37.5°C) with an asexual parasitemia of *P. falciparum*≥500/µLon thin blood film examination. This definition has both a sensitivity and specificity of greater than 90% in children from Manhiça [34]. Children with severe malaria were those presenting with at least one of the following clinical definitions [46]: CM (Blantyre Coma Score≤2), severe anaemia (packed cell volume <15% or hemoglobin<5 g/dL), acidosis or respiratory distress (ARD; lactate>5 mM and/or chest in-drawing or deep breathing), prostration (inability to sit or breastfeed in children old enough to do so based on their age), hypoglycemia (blood glucose<2.2 mM) and multiple seizures (≥2 convulsions in the preceding 24 h). Children with non-severe malaria were those with clinical malaria not showing any of the mentioned signs of severity and able to take oral medication. All severe cases were tested for bacteremia by blood cultures, and children with positive bacteremia were excluded from the study. All severe and uncomplicated cases were reviewed by the study pediatrician to confirm that malaria was the sole or principal cause of the disease. Children with severe disease were admitted at the Manhiça District Hospital and treated with parenteral quinine, and non-severe malaria controls were treated with a combination of oral amodiaquine and sulfadoxine-pyrimethamine (Fansidar®) following national guidelines of Mozambique at the time.

### Collection of parasites

Before treatment, 10 mL of peripheral blood was collected by venipuncture into a tube containing lithium heparin. All blood samples were collected by trained persons in presence of a paediatrician. The amount of blood collected was less than the limit of 3 ml per kg of bodyweight as recommended by ethical guidelines [48]. Blood samples were used for biochemical analysis and blood culture at the study site for clinical management.Biochemical determinations (alanine aminotransferase, bilirubin, lactate and creatinine) and a full blood count were performed for each patient using Vitros DT60 and Sysmex Kx21 analyzers, respectively. Blood samples were centrifuged, IEs were buffy coat depleted, washed three times in phosphate-buffered saline (PBS) and cryopreserved in multiple aliquots in glycerolyte solution at −80°C. Frozen samples were transported to the International Centre for Genetic Engineering and Biotechnology (ICGEB) in New Delhi, and kept in liquid nitrogen until thawed for cytoadherence assays. Ten ml of blood was required for biochemical analysis and blood culture and to provide sufficient parasite aliquots for testing in cytoadherence assays. One aliquot was needed to conduct each of the following assays: 1.) cytoadherence to purified receptors, 2.) cytoadherence to cells expressing receptors CD36, ICAM1, gC1qR, 3.) rosette formation, 4.) platelet-mediated clump formation. Each assay was repeated at least once. Two drops were also spotted onto filter paper (Schleicher & Schuell number 903TM; Dassel, Germany) and used for extraction of DNA for use in PCR analysis.

### Multiplicity of infection

DNA was extracted from blood onto filter papers using the QIAGEN QIAamp DNA Mini Kit. The number of concurrent infections (multiplicity of infection, MOI) was estimated as the highest number of *msp-1* or*msp-2* alleles detected in the sample by PCR [49].

### Thawing and culture of isolates

Cryopreserved *P. falciparum* isolates were thawed by serial dilutions in a gradient of salt solutions following standard methods. Erythrocytes were washed in RPMI 1640 medium containing 2 mM L-glutamine, 25 mM HEPES, 25 mM sodium bicarbonate and 10 µg/mL gentamicin (incomplete RPMI) and cultured at 37°C in complete RPMI (incomplete RPMI supplemented with 0.5% Albumax I and 50 mM hypoxanthine) as previously described [50]. Cultures were monitored by Giemsa-stained thin smears for 18–36 hours and only those that matured to the pigmented trophozoite stage were included in the study. Researchers were blinded to the case-control identification of the isolates.

### Adhesion assays

Measurement of parasite adhesion to purified receptors was done as previously described [51] with slight modifications. Briefly, 25 µL of CD36, ICAM1 (R & D Systems, 20 µg/mL) and gC1qR [18](50 µg/mL) were spotted in duplicate onto 35-mm Petri dishes (BD Labware). Duffy antigen fused to Fc of human IgG (Duffy-Fc [52]; 50 µg/mL) and bovine serum albumin (BSA) (50 µg/mL) were used as negative controls. After overnight incubation at 4°C in moist environment, dishes were blocked with PBS-1% BSA. Parasites were suspended in 1.5 mL adhesion media (RPMI-1640 with 0.5% BSA, pH6.8) at 1% hematocrit and 1% parasitaemia, and incubated for 1 h with the receptors coated on Petri dishes. Samples with less than 1% parasitaemia were enriched using 0.7% Gelatin (Sigma Aldrich) at the pigmented trophozoite stage [53]. After washing with adhesion media till no unbound erythrocytes were seen in the Petri dish under inverted microscope observation, the adherent cells were fixed with 2% glutaraldehyde and stained with Giemsa. The number of adherent IEs per mm^2^ was counted by observation of 10 fields (using 100× objective) under light microscopy. Adhesion to BSA was subtracted to yield specific binding to each receptor.

### Rosetting assays

An aliquot of culture suspension at pigmented trophozoite stage was stained with 20 µg/mL of ethidium bromide in rosetting media (incomplete RPMI supplemented with 10% AB+ human sera). Twenty µL of the suspension at 2% hematocrit was examined by fluorescence and direct light microscopy, and the proportion of IEs in rosettes was measured for each sample in triplicate. Two hundred IEs were scored in each experiment, with the adhesion of two or more uninfected erythrocytes to an IE constituting a rosette. The level of rosetting is reported as the percentage of IEs forming rosettes.

### Preparation of non-activated platelets

Blood was collected from donors by venipuncture in citrate-phosphate dextrose solution (Sigma Aldrich), and centrifuged at 250 *g* for 5 min. Platelet rich plasma was transferred to a separate tube, equal volume of fresh CCDAT buffer (920 mM tri-sodium citrate, 80 mM citric acid, 150 mM glucose, 5 mM adenosine and 3 mM theophylline) was added drop wise and incubated for 15 min. Platelets were collected by centrifugation at 1000 *g* for 20 min and resuspended in CCDAT buffer. Homogenous platelet suspension was transferred to new tubes and centrifuged at 1000 *g* for 20 min. Platelets were finally resuspended in Hanks buffer at 10^8^ platelets per mL, stored in sterile condition at room temperature and used within 4 days of collection. All steps were performed at room temperature. P-selectin (CD62) was not detected on the surface of platelets by flow cytometry [18], indicating that platelets prepared by this way were inactivated.

### Platelet-mediated clumping assays

Parasites were thawed and used for clumping assays as described below. The assays were performed at thawing parasitemias and 2% hematocrit, as well as at 1% adjusted parasitemias (with gelatin enrichment for those samples with less than 1% parasitemia [53]) and 5% hematocrit to discard the confounding effect of different parasitemias between cases and controls [54]. Culture suspensions were stained with ethidium bromide, washed and resuspended in Hanks buffer. Five µL of platelet suspension or Hanks buffer (buffer-control) were added to 45 µL of the IE suspension and the mixture was rotated for 30 min. Five hundred IEs were counted in triplicate for each sampleby fluorescence and direct light microscopy, with the adhesion of three or more IEs constituting a clump. The level of clumping was expressed as percentage of IEs in clumps. Platelet-mediated clumping frequency was defined as the difference between clumping frequency in presence of platelets and in buffer-control.

### Parasite lines

To account for day-to-day variations, adhesion values were normalized using data obtained from parasite lines with known cytoadherence phenotypes: ITG-ICAM1, which binds CD36 and ICAM1, IGHCR14, which binds gC1qR and forms clumps, and R29R+, which form rosettes. These parasite lines were expanded by standard culture and frozen at ring-stage in multiple aliquots previous confirmation of their adhesion profile. Six field isolates were tested per experiment along with control parasite lines used for normalization.

### Definitions and statistical methods

All data collected was entered into the Excel software (Microsoft Co.) and was analysed using the Stata version 9.0 (Stata Corporation). A *P*-value less than .05 was considered statistically significant. Data from cytoadherence experiments were expressed as the mean of duplicate/triplicate measurements. Cytoadherence to purified receptors was considered to be positive if the number of IEs bound per mm^2^to purified receptors coated on Petri dishes was higher than the mean binding plus 2 standard deviations to Duffy-Fc coated Petri dishes. Isolates were considered positive for rosetting if frequency of rosettes was higher than 2% [32] and for platelet-mediated clumping if frequency of clumps was higher in presence of platelets than in buffer-control. Categorical and continuous data were compared between matched case/control pairs by McNemar's chi-squared test and Signtest, respectively. Correlations between variables were assessed by Spearman's rank coefficient. Primary analysis consisted in the comparison of cytoadherence data between parasites isolated from children with severe and uncomplicated malaria. For the secondary analysis, severe malaria cases were grouped according to their criteria for malaria severity, and compared separately to matched controls.

## Supporting Information

Table S1 Platelet-mediated clumping results in assays conducted at 1% parasitemia and 5% hematocrit.
(DOCX)
