# Supplementary material for: Association of Severe Malaria Outcomes with Platelet-Mediated Clumping and Adhesion to a Novel Host Receptor
Source: PLoS One. 2011 Apr 29;6(4):e19422. doi: 10.1371/journal.pone.0019422 (PMC3084855; doi:10.1371/journal.pone.0019422)
Supplement: Table S1 — Platelet-mediated clumping results in assays conducted at 1% parasitemia and 5% hematocrit. (DOCX) [file pone.0019422.s001.docx]

**Table S1.** Platelet-mediated clumping results in assays conducted at 1% parasitemia and 5% hematocrit.

| **Clinical malaria** | **Prevalence, n (%)** | **Intensity, median (IQR)** |
| --- | --- | --- |
| **Uncomplicated (n=36)** | 19 (52.8) | 0.5 (0.0; 4.6) |
| **Severe (n=36)** | 28 (77.8) | 3.4 (0.4; 6.6) |
| ***P*** | **0.029** | **0.024** |
|  |  |  |
| **Uncomplicated (n=26)** | 16 (73.1) | 0.6 (0.0; 5.2) |
| **Prostration (n=26)** | 22(75.9) | 4.0 (0.4; 6.6) |
| ***P*** | 0.109 | 0.122 |
|  |  |  |
| **Uncomplicated (n=13)** | 8 (61.5) | 1.1 (0.0; 4.6) |
| **ARD (n=13)** | 10 (76.9) | 4.0 (0.4; 7.9) |
| ***P*** | 0.317 | **0.038** |
|  |  |  |
| **Uncomplicated (n=11)** | 5 (45.5) | 0.0 (0.0; 3.0) |
| **Severe anaemia (n=11)** | 10 (90.9) | 3.1 (1.0; 6.3) |
| ***P*** | **0.025** | 0.227 |
|  |  |  |
| **Uncomplicated (n=7)** | 4 (57.1) | 0.4 (0.0; 1.4) |
| **Multiple seizures (n=7)** | 5 (71.4) | 3.4 (0.0; 6.5) |
| ***P*** | 0.655 | 0.453 |

ARD: Acute respiratory distress; IQR, Interquartile range.

The analysis was done for 36 pairs of isolates available. Prevalences were compared by MacNemar’s test and intensities by Signtest.
